# Cancer Risk Analysis Based on Improved Probabilistic Neural Network

**DOI:** 10.3389/fncom.2020.00058

**Published:** 2020-07-21

**Authors:** Chaoyu Yang, Jie Yang, Ying Liu, Xianya Geng

**Affiliations:** ^1^School of Economics and Management, Anhui University of Science and Technology, Huainan, China; ^2^Faculty of Engineering and Information Sciences, School of Computing and Information Technology, University of Wollongong, Wollongong, NSW, Australia; ^3^School of Mathematics and Physics, Anhui University of Science and Technology, Huainan, China

**Keywords:** cancer risk analysis, artificial neural network, Naïve Bayes, Markov chain, sparse training

## Abstract

The problem of cancer risk analysis is of great importance to health-service providers and medical researchers. In this study, we propose a novel Artificial Neural Network (ANN) algorithm based on the probabilistic framework, which aims to investigate patient patterns associated with their disease development. Compared to the traditional ANN where input features are directly extracted from raw data, the proposed probabilistic ANN manipulates original inputs according to their probability distribution. More precisely, the Naïve Bayes and Markov chain models are used to approximate the posterior distribution of the raw inputs, which provides a useful estimation of subsequent disease development. Later, this distribution information is further leveraged as additional input to train ANN. Additionally, to reduce the training cost and to boost the generalization capability, a sparse training strategy is also introduced. Experimentally, one of the largest cancer-related datasets is employed in this study. Compared to state-of-the-art methods, the proposed algorithm achieves a much better outcome, in terms of the prediction accuracy of subsequent disease development. The result also reveals the potential impact of patients' disease sequence on their future risk management.

## 1. Introduction

Cancer is a complex health problem worldwide, which is closely monitored by scientists and authorities due to its high mortality rate. In the past decades, the pressure of cancer in public health sectors has gradually increased. A lot of effort has been put into cancer-related studies (Loud and Murphy, 2017), such as patient status monitoring, medical resource allocation, and survivability prediction, to name a few. According to the GLOBOCAN project (Sasikala et al., 2019), there will be more than 14.1 million new cancer-related cases (excluding skin cancer and melanoma) annually, accounting for ~14.6% of global deaths. Even within developed countries, such as the United States, there are more than 1.68 million new patients and 600,000 deaths per year. In particular, Table 1 shows the top eight cancer types from the United States in 2016, while the number of new cases and relevant deaths are also illustrated. For instance, there are about 150,000 new cases diagnosed with breast cancer and around 41,000 deaths, which contribute to a 16.4% ratio between new cases and death numbers. On the other hand, there are ~24,000 new patients and 16,000 deaths related to brain and nervous system cancers, which leads to a significantly high ratio of 67.5%.

**Table 1:** Number of new cancer-related patients and deaths from the United States in 2016.

**Cancer types**	**New cases**	**New deaths**
Digestive system	304,930	153,030
Respiratory system	243,820	162,510
Breast	149,260	40,890
Reproductive system	297,530	57,730
Urinary system	143,190	31,540
Lymphoma	81,080	21,270
Leukemia	60,140	24,400
Brain and other nervous systems	23,770	16,050

As such, the problem of how to monitor and predict cancer-disease development (to reduce its incidence rate) has attracted a lot of attention from different public and private sectors, and has become a major challenge and research focus. The last two decades have witnessed a huge development of computer science and information technologies, which have already taken on an important role in the cancer-related domain. In particular, data mining and machine learning approaches are more regularly employed due to their high performance in simulation and modeling. For example, the work in Heidari et al. (2018) proposed a machine learning based model to identify mammographic image features for short-term breast cancer prediction. Locally preserving projection (LPP) based features were considered, and the experiment was performed using a mammographic dataset collected from 500 women. The result further showed a huge improvement from their work compared to standard methods, such as the Liner Regression and Decision Tree methods. Additionally, a comparison between the Naïve Bayes and K-Nearest Neighbor (KNN) algorithms was provided in Amrane et al. (2018) for breast cancer classification. The experiment was performed using the Wisconsin dataset, while the result showed that KNN outperforms Naïve Bayes with the higher accuracy of 97.51% compared to that of 96.19%. Another breast cancer prediction work has been reported in Jamal et al. (2018), in which authors utilized the hybrid technique of Extreme Gradient Boosting technique and Support Vector Machine. Furthermore, they also applied the Principle Component Analysis (PCA) and K-Means Clustering method to reduce the problem dimensionality. Experimental results illustrated that the hybrid algorithm with a reduced-scale problem indeed improved the prediction performance of diagnosing breast cancer.

However, the majority of the existing research did not address the sequential nature of the disease's development. In other words, less work has been performed to explore the relationship between patients' previous disease and sequential ones. As a result, in this study our research aims to provide new insight into how disease development can be influenced or predicted based on patients' previous medical information. In particular, the Artificial Neural Network (ANN) algorithm is investigated as the optimization tool in our study. ANN is one of the most widely-used techniques for simulation and modeling, due to its ability to learn from complex inputs and to produce accurate outputs. Not surprisingly, we have observed a great number of ANN-based applications in the medical domain. For example, the work from Fakoor et al. (2013) developed a hybrid method by combining ANN with the Support Vector Machine and it was tested on several gene-expression datasets for cancer detection. The results revealed that the ANN-based work outperformed traditional methods via discovering intricate relationships behind risk factors. More recently, a convolutional neural network improvement for breast cancer classification was proposed in Ting et al. (2019). To classify incoming medical images into malignant, benign, and healthy patients, their work performed effectively to localize and identify breast cancer tissue. Other successful implementations of ANN-based models can be found in the survey of Siddiqui et al. (2020).

Despite the general interest in developing the ANN applications, several drawbacks still exist. Specifically, in the context of the disease development, we aim to explore the disease correlation and to identify related risk factors. The majority of traditional ANN applications, however, consider network inputs from the original data directly, while less work has been offered in terms of the input amendment or augment. On the other hand, the standard network training process is usually time consuming, in particular with a large number of inputs. Additionally, as for some real-world scenarios, the generalization performance of the standard ANN is far from being satisfactory.

To this end, in this study we propose a novel hybrid algorithm, based on the idea of Artificial Neural Network, Naïve Bayes, and Markov chain, to address the issue of predicting patients' disease development. In the proposed study, the methods of Naïve Bayes and Markov chain are first applied to estimate posterior possibilities of subsequent development, according to the patient's historical data. The estimation of subsequent possibility is able to establish a relationship model via capturing the underlying correlation of the disease development. Next, estimated possibilities are further leveraged as the input to the neural network, in addition to original inputs. Lastly, we also consider adopting a sparse training strategy for the network training, which is able to optimize the network structure and minimize the training error simultaneously. To the best of our knowledge, this is the first investigation combining the models of Bayesian Network and Markov chain to amend the input of the Artificial Neural Network. The proposed algorithm is further applied to one of the largest cancer-related datasets worldwide, and the comparison with state-of-the-art approaches is also considered.

The rest of this paper is organized as follows. Section 2 provides a review of literature in which several existing research topics are examined, including applications of data-mining techniques on the domain of cancer risk analysis, Artificial Neural Network, Naïve Bayes and Markov chain model. Section 3 provides the basic information about the research background, such as the description of the target dataset used in this study. Section 4 describes the proposed hybrid approach, including the input augment and sparse training. Then, section 5 discusses experiments and comparison results, and finally section 6 concludes the study.

## 2. Literature Review

In this section, we will provide a brief review about existing cancer-related research. Then the fundamental work of Artificial Neural Network, Naïve Bayes and Markov chain model is also provided.

### 2.1. Cancer Risk Analysis

Cancer risk analysis is of great significance to healthcare providers and medical researchers. Several research works have attempted to provide a diverse range of the management and/or prediction strategies for cancer risk analysis. The ultimate goal is to provide precaution for people with a risk, as well as to monitor the disease development (or survivability prediction).

For the risk prediction, the work from Hart et al. (2018) employed a multi-parameterized neural network for lung cancer risk prediction, based on putative risk factors as well as clinical and demographic information. A comparison among Decision Tree, Support Vector Machine, Naïve Bayes, and K-Nearest Neighbors was conducted for a liver-cancer assessment. On the other hand, cancer survivability prediction is also an interesting topic that has been fervently researched throughout the years. The prediction task of cancer survivability is to monitor the possible survivability (the time span) based on the patient's status. For instance, Mayur et al. conducted a study on spinal cord cancer survivability by performing statistical analyses and fitting a Random Forest model (Mayur et al., 2019). The work from Wang et al. (2019) investigated the use of a tree ensemble-based two-stage regression model for advanced-stage lung cancer survival prediction. In addition, a comparison among multiple techniques, including Linear Regression, Decision Tree, Random Forest and Generalized Boosting Machines, and Support Vector Machine, was considered in Sharaf et al. (2015) to predict lung-cancer patient survival.

Despite the great interest in the work of cancer risk and survivability analysis, little research has been done in terms of the relationship between patients' past and current diagnoses. In other words, existing studies fail to address the possibility of subsequent diagnosis, given patients' previous medical conditions. Yet, this research question is of great importance, as it helps in providing prior knowledge of patients' future disease development. To gain an in-depth understanding of potential risk for subsequent diseases also works in increasing the healthcare quality and treatment services (Gupta et al., 2012; Aolin and Maxim, 2017). To bridge this gap, we propose a probabilistic model that takes into account the techniques of the Artificial Neural Network, Naïve Bayes, and Markov chain model.

### 2.2. Artificial Neural Network

The Artificial Neural Network (ANN) is one of the most popular data-mining algorithms, which is capable of responding to complex inputs and generating desired outputs. Due to its satisfactory performance and high accuracy, ANN has found its wide applications in numerous areas, such as pattern recognition, prediction, and statistical simulation, and so on. The most basic computing unit from ANN is the artificial neuron. Those neurons are designed in a similar way to biological neurons within the human brain. In general, input signals are transferred to biological neurons, and then inputs are further processed within their cell bodies. If a certain threshold is reached, neurons are activated to transfer output signals to other neurons. Accordingly, the artificial neuron follows the same procedure of biological neurons: input receiving, threshold activation, and output transferring. Mathematically, suppose the input signal to the *i*-th neuron is a vector of *x*_*i*_, the connection strength to the output is the weight *w*_*i*_, and its bias input is represented as *b*. Given the activation function *f*(·), the output for this *i*-th neuron can be expressed as follows:

(1) $$\begin{array}{r} y=f\left(x_{i}^{T}w_{i}+b\right). \end{array}$$

In real-world applications, the selection of activation function and network structure (the number of hidden layers and/or neurons) is problematic. In general, there is no commonly-accepted formula giving clear insight into how to choose the activation function and/or to determine the network structure. This is usually decided by trial-and-error experiments or cross validation methods. Additionally, after deciding the activation function and network structure, a training process is required to update the internal network weights to minimize the error between the actual network and desired output. Some typical learning algorithms are Back Propagation, Resilient Propagation, and so on.

### 2.3. Naïve Bayes and Markov Chain

Bayesian theory offers a computational framework for estimating the conditional probability, which has proven to be effective for a wide range of applications. Text classification, spam detection, and sentiment analysis are just a few of their popular use cases. Assume that we have one training sample *x* and *n* possible class labels *c*_*i*_ (∀*i*∈*n*). Then the posterior probability (for *x*) of belonging to the *i*-th class [or *prob*(*c*_*i*_|*x*)] can be expressed as:

(2) $$\begin{array}{r} prob\left(c_{i}|x\right)=\frac{prob\left(x|c_{i}\right)prob\left(c_{i}\right)}{prob\left(x\right)},\forall i\in n, \end{array}$$

where *prob*(*c*_*i*_) stands for the class prior probability, *prob*(*x*) is the prior probability of *x*, and *prob*(*x*|*c*_*i*_) denotes the posterior probability of *x* given the condition of the *c*_*i*_ class.

Compared with other classification modes, Naïve Bayes (NB) consumes much less training time, and it can effectively solve small-scale learning problems. For instance, Kim et al. (2018) introduced a Naïve Bayes based text classification in a semantic tensor space model for document representation. URL classification is another classification application of Native Bayes, which is currently of research interest (Rajalakshmi and Aravindan, 2018). In addition, evaluation of a hot-engine test (Fan et al., 2018) and classification of impact damage on a rubber-textile conveyor belt (Andrejiova and Grincova, 2018) are just other use cases that have been investigated using the Naïve Bayes method, respectively.

On the other hand, the Markov chain model is usually utilized to calculate the transition probability from one state to another. In particular, the first order Markov chain operates under the assumption that future states for one particular object (or event) only depend on the current state, but not on other states that occurred before. In other words, let *x*_*i*_ (*i* = 1, 2, ⋯ , *n*) represent a sequence of random variables. Then the probability of moving to the next state (or *x*_*n*+1_) is estimated as:

(3) $$\begin{array}{r} prob\left(x_{n+1}|\left(x_{n},x_{n-1},\cdots \;,x_{1}\right)\right)=prob\left(x_{n+1}|x_{n}\right). \end{array}$$

The Markov chain model proves to be effective in factoring the sequential characteristics of events. Existing applications of the Markov chain model are primarily in the domain of recommendation, speech recognition, and so on. For instance, Ye et al. (2015) and Lassoued et al. (2017) both discussed the use of Markov models in driving route and destination predictions, respectively. Krause and Zhang (2019) proposed a different approach by employing a hierarchical Markov model for short-term behavior prediction. Kurashima et al. (2013) had a slightly different approach when employing not only the Markov Chain model but also a topic model to represent the user interest.

### 2.4. Summary

In this section, we briefly review some existing research on applying the data-mining techniques in the medical domain. Additionally, we also offer a fundamental discussion on three popular methods, including the Artificial Neural Network, Naïve Bayes, and Markov chain model. Based on these three methods, we will then propose a novel prediction algorithm to monitor and predict patients' disease development, which is discussed in the coming sections.

## 3. Study Background

The National Cancer Institute (NCI) established the Surveillance, Epidemiology and End Results (SEER) database in 1973^1^. This incidence database consists of de-identified patient data with different types of cancer diseases. Additionally, for each patient record, there are in total 124 features. These features cover both the demographical and clinical information. For example, demographics information include gender, ethnicity, year of birth, month, and year of diagnosis, age, and marital status of patients at diagnosis. Clinical information includes tumor primary site, tumor marker, tumor size, the types of treatment received, behavior codes, laterality, and histology. In addition, the cancer types involved in the database can be divided into nine categories: breast, colon and rectum, other digestive systems, female reproduction, lymphoid and leukemia, male reproduction, respiratory system, urinary system and other unspecified types. By November 2013, there were more than 1 million data records in the SEER database. Currently, it is the authoritative data source that provides reliable data support for clinical research. A huge number of research efforts have been conducted to utilize this database for different work, such as cancer survival prediction, correlation of medical factors, management of diseases recurrence, and etc.

**Figure 1:**
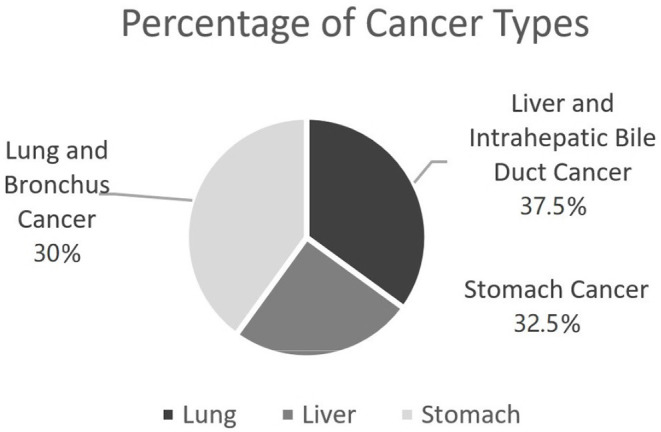
Percentage of selected patients from three cancer types in SEER.

Again, the main purpose of this study is to investigate the possibility of being diagnosed with cancers given a previous medical condition. To model such a disease development, in this study we focus on three types of cancer data from SEER, including lung and bronchus cancer (C1), liver and intrahepatic bile duct cancer (C2), and stomach cancer (C3), respectively. Figure 1 shows the percentage of selected patient samples from three types of cancers.

## 4. Proposed Approach

**Figure 2:**
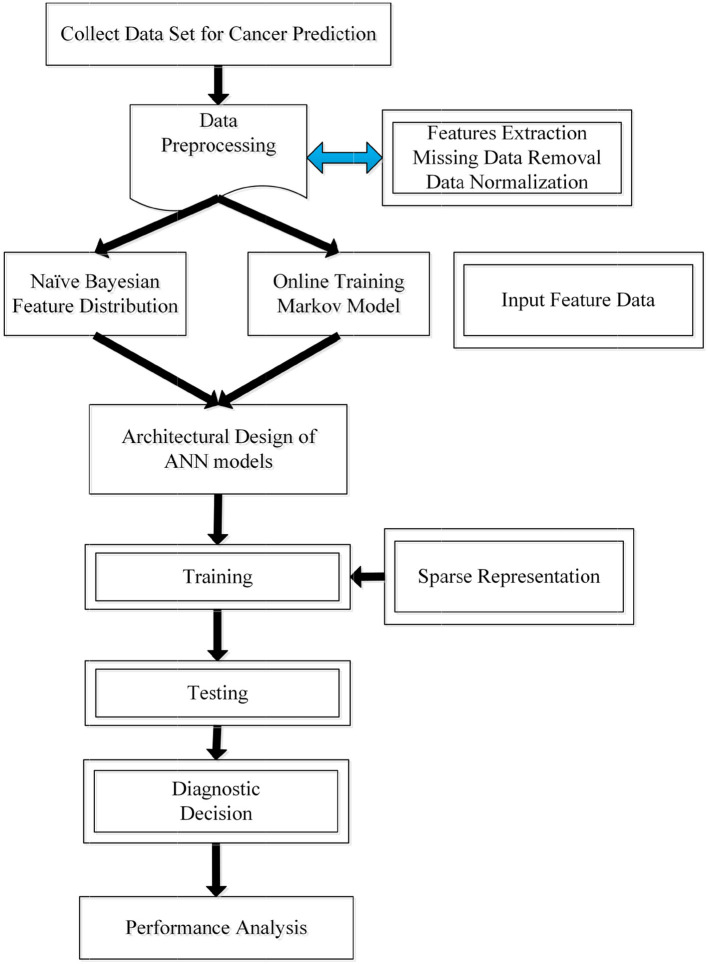
The workflow of the proposed algorithm for predicting patients' disease development.

In this section, we propose a novel prediction algorithm by combining three different methods, including the Bayesian and Markov models, as well as the artificial neural network. Our approach is based on the assumption that the occurrence of a new type of cancer incidence is affiliated with the most recently (or previously) diagnosed cancer incidence, as well as patients' previous clinical details. Toward this end, Naïve Bayesian and Markov chain models are first used to establish the connection between the previous and current incidence, which offers a useful estimation of patient's future status. Then, the output from the two probabilistic models will be cast as the network input for the training process. Additionally, to improve the accuracy and learning efficiency, we further leverage a sparse training strategy for the target network. The pipeline of the proposed algorithm is then illustrated in Figure 2. Next, we will discuss different stages within our proposed algorithm.

### 4.1. Data Pre-processing

To begin with, the first stage is to preprocess the original SEER data to meet certain criteria, such as removal of missing values and data normalization. Among all 124 features, 19 independent features that may have an impact on the cancer prediction tasks were selected, including: gender, race, status, age, primary site, etc. The detail description and value distribution of selected attributes are provided in Table 2.

**Table 2:** Variable descriptions and unique values.

**Variable name**	**Description**	**Unique value count**
PUBCSNUM	Patient's number	1,885,421
SEQ_NUM	Sequence number of all reported incidence	15
YEAR_DX	Year of diagnosis	Continuous
MDXRECMP	Month of diagnosis	12
SEX	Patient's gender	2
MAR_STAT	Marital status at diagnosis	7
RACE1V	Patient ethnicity	30
AGE_DX	Patient's age at diagnosis	Continuous
PRIMSITE	Primary site	51
LATERAL	Laterality	6
FIRSTPRM	First malignant primary indicator	2
HISTREC	Histology	37
GRADE	Histologic grading and differentiation	5
NO_SURG	Reason no cancer-directed surgery	8
EOD10_SZ	Tumor size	Continuous
SS_SURG	Site-specific surgery	30
CSLYMPHN	Involvement of lymph nodes	63
CSEXTEN	Extension of tumor	Continuous
ERSTATUS	Tumor marker 1–breast cancer	5
PRSTATUS	Tumor marker 2–breast cancer	5

Among these features, four of them, namely SS_SURG, CSLYMPHN, EOD10_SZ, and CSEXTEN, contain massive amounts of missing values, ~50% on average. One plausible reason could be the patients' refusal to provide adequate information. On the other hand, due to the evolution of SEER over time, some clinical features have only been collected in recent years. This makes it very impractical to backtrack those new features from previous records. For simplicity, patients' records with missing values will be removed in this study. That is, only completed data samples will be considered.

Next, we find that selected attributes can be divided into discrete and continuous attributes. For discrete attributes, it is easy to process compared to continuous ones. For example, the marital status attribute is divided into seven categories, while the gender one is cast into two categories. By contrast, for continuous data, the minimum-maximum normalization is employed in a way that the values from continuous features will be limited within the range of [0, 1]. Mathematically, let $v_{j}^{p}$ be the value from the *p*-th sample and the *j*-th continuous feature, *min*(*v*_*j*_) and *max*(*v*_*j*_) is the minimal and maximal value of this *j*-th feature from all samples. Accordingly, the normalized value ${\hat{v}}_{j}^{p}$ will be estimated as follows:

(4) $$\begin{array}{r} {\hat{v}}_{j}^{p}=\frac{v_{j}^{p}-min\left(v_{j}\right)}{max\left(v_{j}\right)-min\left(v_{j}\right)}. \end{array}$$

### 4.2. Estimation of Subsequent Disease-Development

In this section, we will discuss the second stage of calculating the possibility of the subsequent disease-development, using the concept of Naïve Bayes and Markov chain model. Suppose we have a set of cancer diagnoses ${\left\{D_{i}^{p},{\left\{v_{ij}^{p}\right\}}_{j=1}^{K}\right\}}_{i=1}^{\tau^{p}}$, where $D_{i}^{p}$ is the *i*-th new type of cancer disease of patient *p*, and $v_{ij}^{p}$ is the *j*^*th*^ feature of the *i*-th new cancer diagnosis of patient *p*, *K* is the number of attributes of the set $\left\{v_{ij}^{p}\right\}$, and τ^*p*^ is the total number of cancer types occurring for patient *p*. Then the research question can be reformulated as follows: given a patient's most-recent cancer diagnosis $D_{i}^{p}$ and the set of patient health profile information at the time of diagnosis ${\left\{v_{ij}^{p}\right\}}_{j=1}^{K}$, the task is to predict the next most likely type of cancer to occur for that patient $D_{i+1}^{p}$. For example, patient *P* had been diagnosed with liver cancer before. In this case, we will investigate the following likelihood of patient *P* having other types of cancers (such as lung or stomach cancer). As a result, mathematically, our goal is to estimate the probability that patient *P* with the *i*-th disease *D*_*i*_ will also develop the (*i* + 1)-th disease *D*_*i*+1_, or the probability $P\left(D_{i+1}^{p}|D_{i},v_{i}^{p}\right)$.

To address the aforementioned problem, we introduce a novel estimation method to calculate the posterior probability based on Naïve Bayes and Markov chain models. More precisely, with Naïve Bayes, we can investigate the dependence of the target variable on a patient's medical condition at the time they are diagnosed with $D_{i}^{p}$. Let $\left\{v_{ij}^{p}\right\}$ be the attribute list of the *p*-th patient. Accordingly, in the Bayes theory, we will have:

(5) $$\begin{array}{r} P\left(D_{i+1}^{p}|v_{i1}^{p},v_{i2}^{p},...v_{iK}^{p}\right)\propto \\ P\left(v_{i1}^{p}|D_{i+1}^{p}\right)P\left(v_{i2}^{p}|D_{i+1}^{p}\right)...P\left(v_{iK}^{p}|D_{i+1}^{p}\right)P\left(D_{i+1}^{p}\right), \end{array}$$

where *K* is the number of attributes. Alternatively, we have

(6) $$\begin{array}{r} P\left(D_{i+1}^{p}|v_{i1}^{p},v_{i2}^{p},...v_{iK}^{p}\right)\propto P\left(D_{i+1}^{p}\right)\overset{K}{\underset{j=1}{\prod }}P\left(v_{ij}^{p}|D_{i+1}^{p}\right). \end{array}$$

The conditional probability $P\left(v_{ij}^{p}|D_{i+1}^{p}\right)$ can be calculated using the Laplace smoothing while avoiding the zero probability:

(7) $$\begin{array}{r} P\left(v_{ij}^{p}|D_{i+1}^{p}\right)=\frac{N\left(D_{i+1}^{p},v_{ij}^{p}\right)+1}{N\left(D_{i+1}^{p}\right)+K}. \end{array}$$

On the other hand, we assume that the next disease relies primarily on the precedent disease, as well as the patient's current status. As such, the Markov chain model is accordingly employed to capture the probabilistic information conveyed by the sequence of diseases, that is identified from patients' medical history. In this study, we consider the first-order Markov model, and accordingly we can estimate the probability of the next disease as follows:

(8) $$\begin{array}{r} P\left(D_{i+1}^{p}|D_{i}^{p},D_{i-1}^{p},...,D_{2}^{p},D_{1}^{p}\right)=P\left(D_{i+1}^{p}|D_{i}^{p}\right). \end{array}$$

Furthermore, the probability of $P\left(D_{i+1}^{p}|D_{i}^{p}\right)$ is calculated as follows:

(9) $$\begin{array}{r} P\left(D_{i+1}^{p}|D_{i}^{p}\right)=\frac{N\left(D_{i+1}^{p},D_{i}^{p}\right)}{N\left(D_{i}^{p}\right)}, \end{array}$$

where $N\left(D_{i+1}^{p},D_{i}^{p}\right)$ is the number of patients with a disease *D*_*i*+1_ occurring right after the disease of *D*_*i*_, and similarly $N\left(D_{i}^{p}\right)$ is the total number of patients with the disease $D_{i}^{p}$.

To incorporate both most-recent diagnosis and the patient's health condition into our proposed model, the above Markov and Naïve Bayes models are combined. Operating under the assumption that the patient's health condition set $v_{i}^{p}$ and $D_{i}^{p}$ are independently conditioned on $D_{i+1}^{p}$, the combination of the two models can be performed using the following approximation:

(10) $$\begin{array}{r} \begin{array}{cc} P\left(D_{i+1}^{p}|D_{i}^{p},v_{i}^{p}\right) & =\frac{P\left(D_{i+1}^{p}|D_{i}^{p}\right)}{C\left(D_{i}^{p},v_{i}^{p}\right)}\frac{P\left(D_{i+1}^{p}|v_{i1}^{p},v_{i2}^{p},...,v_{iK}^{p}\right)}{P\left(D_{i+1}^{p}\right)},\\ =\frac{P\left(D_{i+1}^{p}|D_{i}^{p}\right)}{C\left(D_{i}^{p},v_{i}^{p}\right)}\overset{K}{\underset{j=1}{\prod }}P\left(v_{ij}^{p}|D_{i+1}^{p}\right), \end{array} \end{array}$$

where $P\left(D_{i+1}^{p}|D_{i}^{p}\right)$ and $P\left(v_{ij}^{p}|D_{i+1}^{p}\right)$ can be estimated by the Markov and Naïve Bayes models, respectively, and $C\left(D_{i}^{p},v_{i}^{p}\right)$ is the normalization factor to ensure all probabilities summed to 1.

### 4.3. ANN Training

**Figure 3:**
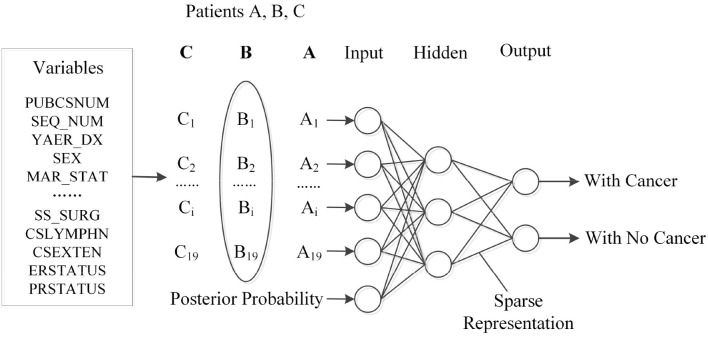
Input structured data for ANN training.

The previous section describes the details about the estimation of subsequent disease development. In the third stage of our proposed algorithm, the output from the previous stage will be cast as the input to feed into a neural network. Figure 3 illustrates the structured input for ANN, while the probability estimation, together with the patient's profile, such as gender and age, are considered as a whole to train the network.

As for the network training process, internal weights will be optimized in a way that the actual network output fits the desired outputs well. Taken as an example, the backpropagation (BP)-based method is a typical way to train ANN via calculating gradients of the output error in relation to network weights. However, the BP-based training could suffer from some drawbacks, such as low convergence and poor generalization capability, in particular with a huge number of input features. In the context of our study, the network has 20 input features, which could be time-consuming for implementing the BP-based training.

To improve the training stability and the fast training speed, we adopt a sparse training strategy in this study, similar to our preliminary work in Yang and Ma (2016, 2019). The general idea is to generate a sparse network structure and to minimize the training error simultaneously. The concept of sparse representation, on the other hand, is under the assumption that a signal can be decomposed into a linear combination of few elementary signals. Consequently, given the target matrix *Y* ∈ ℝ^*M* × *L*^ and a known dictionary matrix $D\in {\mathbb{R}}^{M\times N}$ that contains *N* columns, the sparse representation aims to minimize the solution sparsity and the reconstruction error:

(11) $$\begin{array}{r} X^{*}=\arg\min M\left(X\right)\text{ subjectto }||Y-DX|{|}_{2}⩽\epsilon , \end{array}$$

where M(X) is a measure of the matrix sparsity, $||Y-DX|{|}_{2}$ denotes the reconstruction error, and ϵ is the bound on the error. One simple strategy for estimating M(X) is to consider the *l*_2, 1_-norm of *X*, or $M\left(X\right)=||X|{|}_{2,1}=\underset{q}{\sum }||X_{q}|{|}_{2}$, where *X*_*q*_ denotes the *q*-th row of *X*.

Suppose there are *L* pairs (*x*_*i*_, *y*_*i*_) of inputs *x*_*i*_ and desired outputs *y*_*i*_, while *X* = [*x*_1_, *x*_2_, ..., *x*_*L*_] represents the entire input matrix and *Y* = [*y*_1_, *y*_2_, ..., *y*_*L*_] is the desired output matrix. Additionally, assume that the target network is with a three-layer structure, which consists of *Q*-input, *N*-hidden and *M*-output neurons, respectively. Let $W_{1}\in {\mathbb{R}}^{Q\times N}$ and $W_{2}\in {\mathbb{R}}^{N\times M}$ denote the weight matrices from the hidden and output layer, respectively. As such, the output matrix from the hidden layer (*Z*) can be expressed as:

(12) $$\begin{array}{r} Z=f_{1}\left(XW_{1}\right), \end{array}$$

where *f*_1_(·) is the activation function of the hidden layer, and the *i*-th column from *Z* is in relation to the output of the *i*-th hidden neuron. Furthermore, the actual output from the entire network Ŷ can be written as:

(13) $$\begin{array}{r} Ŷ=f_{2}\left(ZW_{2}\right), \end{array}$$

where *f*_2_(·) is the activation function for the output layers.

The proposed sparse training is then used to optimize the network structure, by selecting the most-important hidden neurons, while minimizing the output error simultaneously. Therefore, the neuron selection process is equivalent to finding a sparse representation for all hidden neurons. Consequently, the sparse training process is then cast as solving the following problem:

(14) $$\begin{array}{r} \min\;||W_{2}|{|}_{2,1}\;\text{subject to }||Ỹ-ZW_{2}|{|}_{2}\leq \epsilon , \end{array}$$

where ||*W*_2_||_2, 1_ is the *l*_2, 1_-norm of the *W*_2_ matrix, $Ỹ=f_{2}^{-1}\left(Y\right)$, and ϵ is the bound on the network error. Note that in the proposed sparse training, we only consider optimizing or sparsifying the weight matrix *W*_2_ between the hidden and output layer. As for the weight matrix *W*_1_ in the previous input-and-hidden layer, we only randomly initialize once during the training and fix them in the subsequent process. The reason is 2-fold: (1) the training performance heavily depends on the output layer, so we focus on the *W*_2_ optimization, instead of both layers; (2) *W*_2_ is trained or adjusted based on the given *W*_1_, as such a random *W*_1_ matrix has a minimal impact on the final output.

### 4.4. Summary

In previous sections, we discuss three different stages from the proposed algorithm. Overall, we apply the Naïve Bayes and Markov chain model to estimate the probability of potential disease development. We then consider this probability result as the additional input, together with other original features, for training a network. At last, to minimize the impact from the huge number of input features, a sparse training strategy is further leveraged to optimize the network structure and minimize the training error simultaneously. Toward this end, Algorithm 1 summarizes the proposed method for investigating the cancer-risk analysis.

**Algorithm 1:** Proposed algorithm for cancer-risk prediction, based on an improved probabilistic neural network.

Stage 1: Data preprocessing, in terms of feature selection, removal of missing records, and perform data normalization.
Stage 2: Calculate the probability based on Equation (10).
Stage 3: Employ the probability result and original input features for network training:
Stage 3.1: Randomly assign weights to the input-hidden layer;
Stage 3.2: Solve the optimization problem in Equation (14) to obtain a spare weight matrix for the hidden-output layer;
Output the trained neural network.

## 5. Experimental Results

This section describes experimental results by applying the proposed algorithm to explore a patient's disease development. The experimental setup and evaluation metrics are presented in section 5.1. In section 5.2, we discuss the probabilities based on their historical information and individual profiles, while the performance of the proposed method is then evaluated in section 5.3.

### 5.1. Experimental Setup

The target dataset includes 10,500 patients with lung cancer, 13,500 with liver cancer, and 12,000 with stomach cancer, respectively, which is a total of 36,000 samples. Each original sample has 19 features, while the majority of chosen features are categorical (or discrete), except for four attributes, such as the patient's age at diagnosis, year of diagnosis, tumor size, and extension of tumor. Again, continuous features will be normalized as described in section 4.1 during the pre-processing stage. We further applied the 3-fold cross validation method to randomly partition the entire dataset into two independent sets: a training and testing set. The size of the training and testing sets in all cases is 75 and 25%, respectively. The training set is used for training the network while the testing set is for evaluation purposes.

Additionally, for the employed neural network, we consider the activation function of the hidden and output layer as the Sigmoid function, which can be expressed as $f\left(z\right)=\frac{1}{\left[1+\exp\left(-z\right)\right]}$ (*z* is an arbitrary input). The layer between the input-and-hidden is initialized with random weights in the range [-1, +1]. The number of hidden neurons is set as 64. To solve the optimization problem in Equation (14), the orthogonal matching pursuit (OMP) algorithm is employed^2^, which first measures the similarity between the residual error and the neuron outputs, and then selects the neuron that minimizes the residual error at each iteration. To halt the OMP solver, the termination criterion is set either when the maximal iteration (*K*) is reached or when the value of $\frac{||\epsilon_{k}-\epsilon_{k-1}|{|}_{2}^{2}}{||\epsilon_{k}|{|}_{2}^{2}}$ is less than a threshold α, where ϵ_*k*_ is the output error at the *k*-th iteration, and α is a user-defined value. Lastly, the following metrics are employed to evaluate the performance:

(15) $$\begin{array}{r} Recall=\frac{TP}{TP+FN}, \end{array}$$

(16) $$\begin{array}{r} Precision=\frac{TP}{TP+FP}, \end{array}$$

(17) $$\begin{array}{r} F1\;Score=2\times \frac{Precision\times Recall}{Precision+Recall}, \end{array}$$

where TP denotes the true positive rate, FN is false negative rate, and FP represents the false positive, respectively.

### 5.2. Probabilities for Disease Prediction

In this section, we discuss the result of patients' disease probabilities using their previous medical information. As mentioned before, this temporary result, obtained from Naïve Bayes and Markov chain model, will be cast as the input to the subsequent network training. Therefore, an accurate estimation of posterior probabilities will certainly enhance the network performance. Before we discuss the result, the detail of forming the patients' historical information is provided first. Again, we are interested in three types of cancers in this study: lung, liver, and stomach cancer. As such, the entire dataset is grouped by the patient ID. These records are further sorted based on the date of disease diagnosis, while records are indexed from 0, and the maximum number of incidences from a patient is five. Note that some patients could have the problem of recurrence, thereby leading to more than three records. Next, the following procedure is considered:

1. If the patient only has one type of cancer, then her/his record is added directly to the final dataset;
2. If the patient has a recurrence, then records with same type are merged by maintaining only one sample with the latest date of diagnosis.

Through the aforementioned process, redundant patients' records are removed, and the sequence of disease development is accordingly established for the following calculation. Lastly, the estimation result of posterior probabilities, given the patients' previous information, is presented in Table 3.

**Table 3:** Patient's conditional probabilities.

	**$N(D_{i+1}^{\;p},D_{i}^{\;p})$**	**$N(D_{i}^{\;p})$**	**$P(D_{i+1}^{\;p}|D_{i}^{\;p},v_{ij}^{\;p})$**
$D_{i+1}^{p}=C1,D_{i}^{p}=C2$	10,864	24,085	0.4346
$D_{i+1}^{p}=C3,D_{i}^{p}=C2$	13,726	26,421	0.5756
$D_{i+1}^{p}=C2,D_{i}^{p}=C1$	6,821	16,548	0.3753
$D_{i+1}^{p}=C3,D_{i}^{p}=C1$	10,889	17,009	0.6588
$D_{i+1}^{p}=C2,D_{i}^{p}=C3$	2,112	6,981	0.3631
$D_{i+1}^{p}=C1,D_{i}^{p}=C3$	3,219	6,811	0.6312

*Note that we label lung and bronchus cancer as C1, liver and intrahepatic bile duct cancer as C2, and stomach cancer as C3, respectively*.

From the results presented in Table 3, there indeed exists some connection between patients' disease development. For instance, we observe that the probabilities from 50% of cases (three out of six) have exceed 57%, which indicates a potential correlation among different diseases. The highest value is found from patients with a type of lung cancer (C1), who have more than a 65% possibility to develop stomach cancer (C3). On the other hand, for patients who had stomach cancer (C3) previously, the chance is much lower (only about 36%) to develop liver cancer (C2). This preliminary result will then be cast as the input for the subsequent network training, while the comparison with other methods is discussed in the next section.

### 5.3. Comparison With Other Training Algorithms

Note that again in our proposed algorithm, the main contribution is 2-fold: (1) introducing the technique of Naïve Bayes and Markov chain models to estimate the posterior possibilities; (2) employing the sparse training strategy for the network training. As such, the following experiments are designed to evaluate the effectiveness of both the possibility result and the sparse training.

To begin, we consider comparing the performance of the standard ANN, combination model with Bayes and Markov (labeled as CBM), and the proposed models on the training and test set, respectively. Note that in the standard ANN, original features are directly fed into the network, while no additional input is considered. In the CBM method, the estimation for potential disease is considered but no additional neural network is attached. We run the experiments 10 times, and average results are summarized and presented in Tables 4, 5, respectively.

**Table 4:** Comparison of evaluation metrics from the training dataset.

	**ANN (%)**	**CBM (%)**	**Proposed (%)**
Overall accuracy	73.55	76.07	75.63
RECALL (C1)	98.64	98.84	98.05
RECALL (C2)	86.29	87.48	87.42
RECALL (C3)	46.14	49.61	50.92
Precision (C1)	69.28	72.49	71.73
Precision (C2)	59.72	63.02	63.07
Precision (C3)	89.42	91.76	90.84
F1 score (C1)	80.46	83.74	82.57
F1 score (C2)	73.81	76.49	75.38
F1 score (C3)	58.21	61.18	60.72

*Again, the labels of C1, C2, and C3 represent the lung and bronchus, liver and intrahepatic bile duct cancer, and stomach cancer, respectively*.

**Table 5:** Comparison of evaluation metrics from the test dataset.

	**ANN (%)**	**CBM (%)**	**Proposed (%)**
Overall accuracy	68.78	70.63	72.47
RECALL (C1)	77.44	77.34	78.11
RECALL (C2)	81.82	82.03	83.79
RECALL (C3)	58.19	65.83	66.64
Precision (C1)	65.37	67.12	69.75
Precision (C2)	63.81	65.72	67.91
Precision (C3)	75.93	77.56	78.37
F1 score (C1)	78.34	75.39	78.95
F1 score (C2)	65.23	65.17	65.06
F1 score (C3)	52.89	59.52	63.63

*Again, the labels of C1, C2, and C3 represent the lung and bronchus, liver and intrahepatic bile duct cancer, and stomach cancer, respectively*.

When it comes to the training performance, we realize that the probability estimation for patients' status indeed helps in boosting the accuracy. For instance, both the CBM and proposed algorithms achieve better training outcome compared to that of the standard ANN method. Again, the major difference among the three methods lie in the input; the results suggest that the additional estimation of patients' status (based on their previous information) is capable of providing useful information that facilitates the subsequent ANN training.

On the other hand, we also observe the best generalization performance of the proposed algorithm from Table 5. The results from the test dataset indicate that the ANN performs the worst, while the CBM method comes second. However, we also notice that the training performance of the proposed algorithm (75.63%) is slightly lower than that of CBM (76.07%) from Table 4. The reason could be the overfitting of CBM to the training data, while the employed sparse neural network helps in improving the testing accuracy while avoiding the overfitting. As a result, the experimental results confirm the advantage of both the additional input from posterior probability and the sparse training in the proposed algorithm.

Next, the performance of our algorithm is compared with conventional methods, and the aim is to evaluate the effectiveness of the proposed method. More precisely, the Support Vector Machine and Random Forest algorithms are included in this paper for comparison purposes:

1. Support Vector Machine (*SVM*) is one of the most popular kernel-based approaches, which has been demonstrated to perform well in various applications (Sharaf et al., 2015). Usually, the decision boundary formed by SVM is constructed by finding a hyperplane that achieves the maximum separation between classes. In this study, the implemented SVM is with the radial basis function (RBF) kernel, while the penalty parameter *C* of the error term is set as *C* = 0.01, and the Kernel coefficient γ is set as γ = 0.1;
2. Random Forest (*RF*) is one typical ensemble method, which establishes a forest by constructing a collection of element decision trees (Mayur et al., 2019). For each element tree, RF allows them to randomly choose a subset of features from the entire set, which enhances its flexibility and stability. Key hyperparameters within RF include the number of trees in the forest (*n*_*estimators*), the maximum depth of a tree (*max*_*depth*), and the number of features for splitting (*max*_*features*). In this study, we adopt the following: *max*_*depth* = 5, *n*_*estimators* = 10, and $max\text{\_}features=\sqrt{n\text{\_}features}$ (where *n*_*features* is the number of total features).
3. Extreme Learning Machine (*ELM*) is one typical network training algorithm, which initializes the network weights randomly and then update the weight matrix in the output layer based on a least-square model (Wang et al., 2020). Experiments have shown the advantage of ELM to have easy implementation and better generalization ability, compared to the traditional backpropagation training algorithm. As such, ELM is introduced to make a comparison with the proposed algorithm with a typical three-layer network, while the number of hidden neurons is set as 64.
4. The weighted association rules algorithm (*WCBA*) aims to generate association rules by combining a new attribute evaluation and prioritization techniques (Alwidian et al., 2018). More precisely, domain knowledge was employed to identify attributes with high significance. Then the statistical harmonic mean (HM) measurement was introduced to prioritize generated rules at the pruning and generation phases. Experimental results show its effectiveness by comparing existing rule-based classification methods.

**Figure 4:**
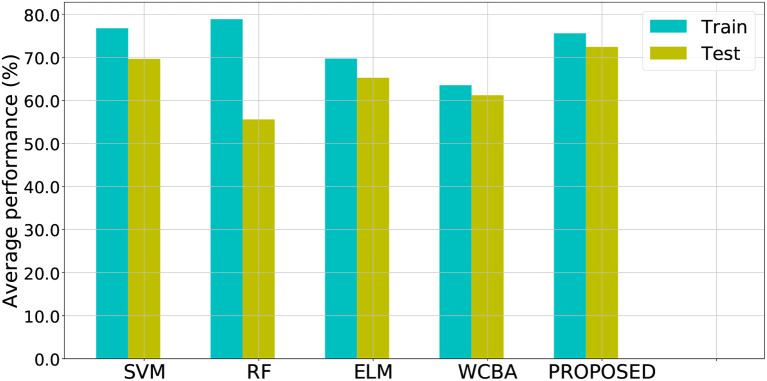
Average training and testing accuracy obtained from different algorithms for prediction.

**Figure 5:**
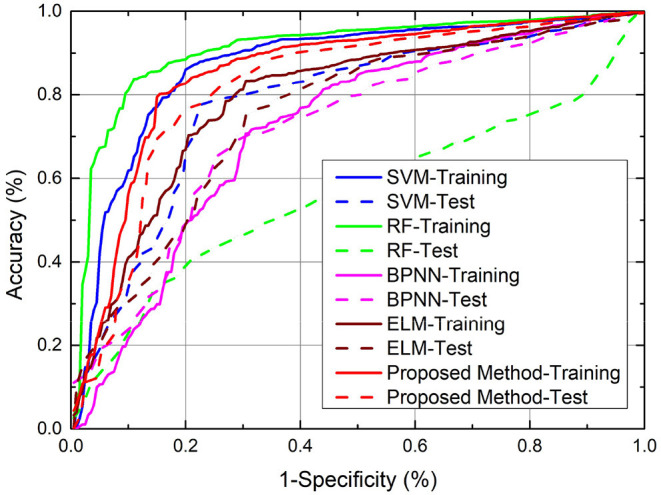
Comparison of classification accuracy (ROC curves) from various methods.

Note that for SVM, RF, ELM, and WCBA, their inputs are from original data directly, without the additional posterior possibility information. We ran the experiments 10 times to obtain the average performance. As a result, both the training and test classification accuracy from different methods are shown in Figure 4, and the relevant ROC curves are also shown in Figure 5. Although the SVM and RF method have performed better in the training cases, they seem to have problems with overfitting. In particular, the RF method leads to the highest accuracy of 78.93% from training, but with a poor testing accuracy of 55.62%. A similar problem was observed in the SVM method. By contrast, compared to those standard algorithms, the proposed approach achieves a notable improvement in terms of testing accuracy. For instance, our method leads to the best testing result of 72.47%, which is significantly better than the accuracy of SVM (69.70%), RF (55.62%), ELM (65.31%), and WCBA (61.25%), respectively. Overall, it is empirically confirmed that the proposed method outperforms existing training methods by improving the generalization capability.

## 6. Conclusions

Understanding patients' cancer risks, using their historical medical information, is of significant interest in healthcare management. There are still many challenges that remain, including high dimensionality and the heterogeneous structure of data. In this study, a novel algorithm based on the improved probabilistic neural network is proposed, with the ultimate aim of providing decision support for cancer-risk management. The main contribution of our work is 2-fold: (1) we factor the sequential state information with the first-order Markov chain and Naïve Bayes models; this sequential information is then represented as the posterior probability and cast as the additional input for training the neural network; (2) we consider adopting the sparse training strategy to boost the network performance, which is able to optimize the network structure and minimize the training error simultaneously. We test our method using one of the largest cancer-related datasets worldwide. Experimental results suggest that our proposed algorithm exhibits some potential for accurate predictions, compared to other conventional methods. Future work can then apply our method in a broader range of applications, or to develop more sophisticated probability-based neural networks.

## Data Availability Statement

The datasets analyzed for this study can be found in the link of https://seer.cancer.gov/.

## Author Contributions

CY: conceptualization, methodology, software, validation, investigation, visualization, and writing original draft. JY: software, writing—review and editing, and supervision. YL: software, visualization, and writing—original draft. XG: writing—review and editing, validation, and visualization. All authors contributed to the article and approved the submitted version.

## Conflict of Interest

The authors declare that the research was conducted in the absence of any commercial or financial relationships that could be construed as a potential conflict of interest.

## Footnotes

^1^Available online at: https://seer.cancer.gov.

^2^Available online at: https://scikit-learn.org.
